# Emotional and autoimmune triggers in Takotsubo cardiomyopathy: A case report of a young female with systemic lupus erythematosus

**DOI:** 10.1097/MD.0000000000047097

**Published:** 2026-01-09

**Authors:** Ziad W. Elmezayen, Alaa Zayed, Enas Samara

**Affiliations:** a Faculty of Medicine, Kafr Elsheikh University, Kafr Elsheikh, Egypt; b Department of Medicine, Faculty of Medicine and Health Sciences, An-Najah National University, Nablus, Palestine.

**Keywords:** apical ballooning, cardiac imaging, emotional stress, lupus flare, myocardial dysfunction, systemic lupus erythematosus, Takotsubo cardiomyopathy

## Abstract

**Rationale:**

Takotsubo cardiomyopathy (TCM), also known as stress-induced cardiomyopathy, is an acute heart condition that mimics acute coronary syndrome and usually affects postmenopausal women. In young patients with autoimmune disorders like systemic lupus erythematosus (SLE), it is uncommon and difficult to diagnose. This case report emphasizes emotional stress and autoimmune flare as co-triggers of TCM and contributes to the limited literature on such presentations.

**Patient concerns:**

A 27-year-old woman with SLE presented with acute chest discomfort, palpitations, and shortness of breath after her father’s sudden death. She also mentioned weariness, joint discomfort, and anxiety, all of which are typical of a lupus flare.

**Diagnoses:**

Electrocardiography revealed sinus tachycardia as well as ST-segment increases in the anterior leads. Troponin I and NT-proBNP levels were found to be increased. Coronary angiography revealed normal coronary arteries, while echocardiography revealed apical ballooning of the left ventricle, confirming the diagnosis of TCM.

**Interventions:**

The patient was given intravenous methylprednisolone for lupus flare management, as well as metoprolol, intravenous fluids, hydroxychloroquine, and lisinopril after stabilization. Emotional support and education on stress management were also provided.

**Outcomes:**

The patient’s cardiac function and lupus activity improved significantly. She was discharged in stable condition after 6 days and remained asymptomatic 3 months later, with no return of cardiovascular symptoms and complete echocardiographic resolution.

**Lessons:**

This case reinforces the importance of evaluating TCM in young SLE patients with acute chest pain, particularly when emotional stress is involved. Excluding coronary artery disease is essential, and effective management requires a multidisciplinary approach that treats both cardiac and autoimmune components. Preventing recurrence demands integrating emotional and psychological support into the care of chronically ill individuals.

## 1. Introduction

Takotsubo cardiomyopathy (TCM), also known as stress-induced cardiomyopathy or left ventricular apical ballooning syndrome, was first described by Japanese physicians in 1990.^[1]^

Though initially thought to be a rare phenomenon, according to recent data, TCM is estimated to account for 1% to 2% of all cases of suspected myocardial infarction, with a peak incidence during summertime.^[2]^

Exposure to an emotional or physical stressor typically incites TCM, with the reception of bad news persisting as its most common and recognizable trigger. However, numerous other inciting events have been reported.^[3]^ The etiology of TCM is not well understood. Current pathophysiologic hypotheses consider the hypothalamic–pituitary–adrenal axis and sudden catecholamine excess which results in elevated blood pressure and catecholamine toxicity in the heart, particularly affecting myocardial cells in areas with high adrenoreceptor expression, Additionally, the loss of estrogen may amplify these responses, making postmenopausal women more susceptible to TCM.^[4]^

The electrocardiogram (ECG) might show ST-segment changes or T-wave inversions.^[5]^ Typically, troponin is elevated.^[6]^ Diagnostic investigations include electrocardiography, coronary angiography, and cardiac MRI.^[5]^ Treatment involves monitoring and treatment of potential complications. Hospital mortality rates are approximately 2%.^[7]^

We report a case of a young female patient with a history of Systemic lupus erythematosus (SLE) who presented with severe chest pain, shortness of breath, and palpitations beginning after a stressful family event, along with fatigue, joint pain, and anxiety, who was later found to have TCM.

## 2. Case presentation

A 27-year-old female patient presented to the emergency department with severe chest pain, palpitations, and shortness of breath. The symptoms developed after an emotional event that she experienced: her father died. She also complained of generalized fatigue, joint pain, and anxiety. The patient was diagnosed with SLE 3 years ago and managed with hydroxychloroquine (200 mg daily oral) and corticosteroids. She had no history of cardiovascular disease, diabetes, or hypertension. Family history was unremarkable.

On physical examination, the patient was anxious and distressed. Blood pressure was 90/60 mm Hg, heart rate of 125 bpm, respiratory rate of 22 breaths per minute, and oxygen saturation of 96% on room air. Examination revealed constitutional symptoms, including fatigue and a recent 5-kg weight loss. She had joint pain in the hands and knees suggestive of a lupus flare, and occasional headaches without dizziness or syncope.

**Figure 1.**
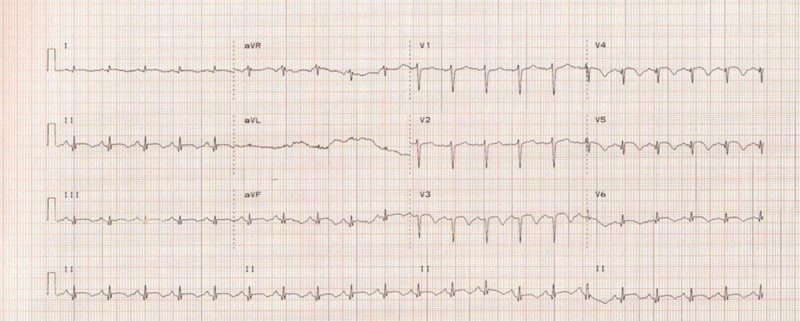
ECG at initial presentation revealed ST elevation at the anterior leads with negative T-waves. ECG = electrocardiogram.

**Figure 2.**
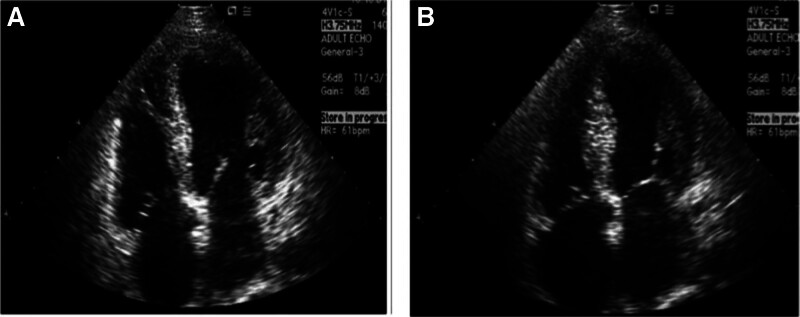
Initial echocardiography showing apical ballooning at diastole (A) and at systole (B) of apical 4-chamber view.

Electrocardiography showed sinus tachycardia and ST-segment elevations in the anterior leads (Fig. 1). Troponin I was 1.5 ng/mL, and NT-proBNP was 800 pg/mL. Coronary angiography demonstrated normal coronary arteries, which rules out ischemic heart disease. Transthoracic echocardiography indicated apical ballooning of the left ventricle, consistent with TCM (Fig. 2).

Laboratory investigations revealed normal renal and electrolyte profiles, trace proteinuria with little hematuria on urinalysis, and mildly decreased hemoglobin and hematocrit. Active lupus was confirmed by the autoimmune panel’s positive results for antinuclear antibodies (ANA) and high anti-double-stranded DNA (anti-dsDNA) antibodies, as shown below in (Table 1).

**Table 1:** Laboratory investigations at admission.

Autoimmune panel			
ANA	Positive (1:160)	Anti-Smith antibodies	Positive
Anti-double-stranded DNA antibodies	Elevated	–	–
Complement levels			
C3	70 mg/dL (low)	C4	10 mg/dL (low)
CBC			
Hemoglobin	12.0 g/dL	Hematocrit	36%
Urinalysis			
Protein	Trace amounts	Red blood cells	2–3 per HPF

ANA = antinuclear antibodiesm, CBC = complete blood count, HPF = high power field.

The patient was diagnosed with TCM during a lupus flare, which was most likely induced by emotional stress. For the lupus flare, management included intravenous methylprednisolone (40 mg), beta-blocker medication with oral metoprolol (25–50 mg titrated), intravenous fluids, continuation of hydroxychloroquine, and commencement of lisinopril (5 mg orally) after hemodynamic stabilization. Her symptoms improved markedly, cardiac markers returned to normal, and resolution of apical ballooning on the follow-up echocardiography. She was discharged after 6 days in stable condition, after receiving stress management and medication adherence education. At 3-month follow-up, the patient remained asymptomatic, with steady lupus activity and no recurrence of cardiovascular problems.

## 3. Discussion

This case presents a rare and diagnostically challenging intersection of TCM and SLE in a young woman with no prior cardiovascular disease. TCM is most commonly described in postmenopausal women following emotional or physical stress and is characterized by transient regional systolic dysfunction of the left ventricle in the absence of significant coronary artery disease.^[8]^ In contrast, SLE is an autoimmune disorder predominantly affecting young females and associated with widespread systemic inflammation, including cardiovascular complications.^[9]^

The patient, a 27-year-old woman with a 3-year history of SLE, presented with acute chest pain, palpitations, and shortness of breath following the sudden emotional trauma of losing her father. These symptoms were accompanied by systemic features of a lupus flare. ECG findings of ST-segment elevation in anterior leads and elevated troponin I and NT-proBNP. In isolation, these findings could suggest acute coronary syndrome (ACS), lupus myocarditis, or even pulmonary embolism. However, coronary angiography revealed normal coronary arteries, and echocardiography demonstrated classic apical ballooning, supporting a diagnosis of TCM. These findings underscore the need for a high index of suspicion for TCM in young patients with autoimmune diseases, especially when emotional stressors are evident.

TCM is widely recognized as a stress-induced cardiomyopathy, frequently triggered by sudden emotional or physical stress.^[3,10]^ In this case, the acute grief from her father’s death likely served as the primary emotional trigger, consistent with known psychogenic precipitants of TCM. However, in SLE patients, autoimmune mechanisms may also play a role in myocardial susceptibility.^[10]^ Chronic systemic inflammation, immune complex deposition, and cytokine-mediated endothelial dysfunction may enhance the vulnerability of myocardial tissue to stress-induced injury.^[11]^ This dual mechanism, emotional and autoimmune, likely acted synergistically in this patient. A previous study of TCM and autoimmune disorders by kouhanjani et al showed that SLE is the second most common cause of TCM in autoimmune disorders.^[10]^

One of the most important diagnostic challenges in this case was distinguishing TCM from lupus myocarditis and ACS, which can present similarly. Lupus myocarditis may present with chest pain, ECG changes, and raised troponin levels, often in the setting of active disease.^[12]^ However, myocarditis typically shows global hypokinesis,^[12]^ rather than the regional apical ballooning pattern seen on echocardiography in TCM.^[13]^ Troponin levels can be elevated in TCM, ACS, and lupus myocarditis, but the patterns differ. In TCM, the rise in troponin is usually modest compared to the significant ECG changes and heart wall motion abnormalities. In contrast, ACS typically shows a much higher troponin elevation that closely reflects the extent of heart muscle damage.^[14]^ Lupus myocarditis can also cause a troponin increase, but it is often less pronounced and usually occurs alongside elevated inflammatory markers.^[12]^

Furthermore, the normalization of ventricular function on follow-up echocardiography supports TCM, which is known for its reversibility.^[15]^ Coronary angiography played a critical role by ruling out obstructive coronary disease, thereby fulfilling one of the major criteria for TCM diagnosis.^[3]^

This case required a multidisciplinary approach due to the overlapping cardiac and rheumatologic aspects. The patient received high-dose intravenous methylprednisolone to manage the lupus flare, which was critical in controlling systemic inflammation. Beta-blockers (metoprolol) were administered to reduce myocardial oxygen demand and blunt the effects of catecholamines, aligning with TCM management guidelines.^[16]^ Intravenous fluids were used cautiously to support blood pressure, and lisinopril was introduced after stabilization for its cardioprotective and renal effects. Continuation of hydroxychloroquine helped maintain long-term lupus control. Hemodynamically stable TCM patients without complications should be monitored in cardiology. Short-term use of dual antiplatelet therapy (DAPT), anticoagulants, beta-blockers, statins, and ACE/ARBs may reduce complications, but DAPT should be stopped at discharge if TCM is confirmed.^[16]^ Long-term benefit in preventing recurrence remains unclear.

Prognosis and risk of recurrence the prognosis of TCM is generally favorable, with most patients recovering normal cardiac function within days to weeks.^[16]^ A 10-year study by Lau et al showed that 7.5% had recurrence and 16.2% died. Older age, male sex, diabetes, lung, and kidney disease increased the risk. Beta-blockers improved survival, while ACE inhibitors/ARBs had no effect.^[17]^ In this case, echocardiographic follow-up showed complete resolution of wall motion abnormalities, and the patient remained symptom-free 3 months post-discharge. In patients with SLE, continued disease control and stress management are crucial to minimizing recurrence risk.

This case highlights several key lessons. First, TCM should be considered in SLE patients presenting with acute chest pain, especially if emotional stress is involved. Second, reliance on ECG and troponin levels alone may be misleading; imaging and coronary angiography are essential for accurate diagnosis. Third, emotional stress and autoimmune inflammation can act synergistically to precipitate TCM. Fourth, management requires addressing both cardiac and systemic lupus components, emphasizing the need for collaborative care between cardiology and rheumatology teams. Finally, the case underscores the necessity of incorporating mental health evaluation and support into the care of chronically ill patients.

## 4. Conclusion

This case demonstrates a rare but clinically important overlap between Takotsubo cardiomyopathy and SLE in a young female patient. The interplay of emotional trauma and autoimmune inflammation likely triggered transient left ventricular dysfunction, mimicking ACS. A comprehensive diagnostic approach, timely cardiac imaging, and appropriate management of both the cardiac and autoimmune components led to a full recovery. This case underscores the need for clinical vigilance and a multidisciplinary strategy in managing complex cases with overlapping etiologies.

## Author contributions

**Conceptualization:** Ziad W. Elmezayen, Alaa Zayed, Enas Samara.

**Supervision:** Alaa Zayed.

**Writing – original draft:** Ziad W. Elmezayen, Alaa Zayed, Enas Samara.

**Writing – review & editing:** Ziad W. Elmezayen, Alaa Zayed, Enas Samara.
